# Treatment and Filling of the Nerve Canals in Teeth

**Published:** 1885-03

**Authors:** John Stirling


### THE DENTAL REGISTER.

VOL. XXXIX.] MARCH, 1885. [No. 3.

### COMMUNICATIONS.

### Treatment and Filling of the Nerve Canals in Teeth.

BY JOHN STIRLING, L.D.S., ENG.

A paper read before the Odonoto-Chirurgical Society of Scotland.

We are gradually progressing in the proper treatment of aching teeth. It is not very many years since the filling of roots was an operation we only heard mentioned, and never saw accomplished, and when it was the almost universal practice to fill teeth over a devitalized nerve, without any attempt to remove it. Co-existent with that was the now old-fashioned operation of rhizodontrophy. We have not yet arrived at perfection in the treatment of roots, but we have improved very much upon that; which, I hope, will be shown in the discussion of the subject here to-night.

In the first place, I will consider the treatment of the teeth preparatory to filling their roots, and I will take the simplest and easiest of cases first--that is, devitalizing and extirpating the nerves of the teeth.

In destroying a tooth pulp, when arsenic is employed, it should not be used as we get it from the chemist's shop, but should be triturated in a small mortar, moistened with water, from half an hour to an hour, and I prefer to use it with notbing but carbolic acid added to it. I usually leave the extirpating of the pulp till about four weeks after applying arsenic. The dressing is removed after twenty-four or forty-eight hours, and the pulp chamber well opened into, cutting out at the same time the bulbous portion of the pulp, after which it may safely, or rather advantageously, be left alone for a month or more.

If any attempt be made to remove the nerve on the same day, or within a few days of removing the dressing, it must in most cases cause considerable pain to the patient. At the end of four weeks the pulp should be cleared out of the canals. I use these instruments as nerve extractors. You will see they are round, and slightly barbed or roughened, with a sharp sculptor.

If, when clearing out the nerve, there is found to be still some vitality at the far end of the canals, drenching it well with carbolic acid, and careful manipulating with the nerve extractor, will get it out painlessly. Where there has been little or no putrefaction of the pulp, further than extirpation of the pulp, the canals require no preparation or treatment. I very rarely open a canal with broach or drill to the apex of the root. The canals may be filled immediately after removing the nerve.

I have not had any experience in treating a devitalized nerve with tannic acid, and then extracting it entire, but if that can be done painlessly, it is to be commended. Such a tooth could be finished in two or three days from the time its treatment was commenced. In upper front teeth, after devitalizing the nerve, when I put in a temporary stopping, I often, at the end of a month, remove that nerve entire, with one draw of the instrument, without pain to the patient. But if there has been the slightest gangrene of the nerve, a temporary stopping is inadmissible; the cavity must be left open.

Where I find inflammation of a nerve, caused perhaps by a stopping being too near to it, if it be at all severe, or especially if it has extended to the periosteum, I devitalize and eradicate the nerve in the manner which I have just described. I have no faith in capping such a nerve; an accidental exposure, of course, I always cap, but any tooth pulp that has once been very decidedly inflamed, and has once given severe toothache, will never return to its normal state; cap and stop with non-conducting material as you will, the pulp will always remain in a chronic state of inflammation. The capping may stop ordinary toothache, but the tooth will be always sensitive to thermal changes, causing occasional discomfort to the patient, which may go on from a few weeks to a year, or longer; finally that passes away, the tooth remains quiet for a short time, and the patient can take cold water freely without hurting it. But it is only preparing for more serious trouble, which comes soon or late, but generally soon, and the stopping has to be removed, and the capping ignominiously turned out.

There are cases where the nerve seems to shrivel and dry up, leaving comparatively clean canals, and which give little or no further trouble. But these cases are exceptional and usually exceptional not in the case of the tooth, but of the patient, whose teeth pulps generally all follow the same course.

But we have a very different state of things where we find a tooth, in the canals of which putrefaction has been going on for some time, causing periodontitis or alveolar abscess. The most part of the work of stopping that tooth will be in cleaning the nerve canals, and bringing the periodonteum into a healthy condition. In a case of that kind, where there is, say, acute periostitis, caused by unclean nerve canals, we all know the symptoms presented, and can almost tell it at a glance. The first thing to do, is to open the pulp cavity well up. If the tooth is very painful to the touch of the finger, we may not be able to do this effectually. But if we are to give relief from toothache, it must be opened enough to allow us to pass one of these instruments a short distance into, at least, one of the canals; and we can generally manage that with a sharp excavator, a gentle touch, and a good light. When, however, we can cut freely without hurting, we are able to do a little cleaning to the canals, but it must be done carefully, and they must be wiped out, not washed out. A syringe and water at this time should not be used, because to employ it with effect, it must be used with force, and to force water into the canals, would probably drive through their apical foramina something septic or mephitic, that would rather increase the inflammation that we are trying to subdue.

I usually put a little bicarbonate of soda into the cavity of the tooth, moistened with one drop of water, pass an instrument into the canal, and work it a few times up and down and against its sides, and then let the patient rinse the mouth.

If that fails to give relief, try carbolic acid, with a very little iodoform in it, pushed gently into the canals. If part of the nerve is still alive and highly inflamed, iodoform with carbolic acid, and not arsenic, should be used. Failing to give any immediate relief, the patient may be directed to hold cold water in the mouth five or six times during the day for a quarter of an hour each time. A saline purgative is also a good thing in such a case. In any case, I never recommend the extraction of a tooth where there is acute periostitis, before suppuration has taken place, because the tooth (though sometimes a little loose, from swelling of the periosteum) then clings harder to its socket; it would be more readily broken in the attempt to extract it, the operation would be more painful; and when successfully extracted, pain would probably continue in the socket for some time after.

Let us suppose, now, that the disease, before we saw it, had gone a stage farther, and suppuration had taken place. If it were a lower molar tooth, with the nerve canals difficult of access, and especially if the patient had reached the shady side of middle age, I would recommend its extraction. But if it were a young tooth, with the canals wide, and easy of access, or if it were any other tooth than a lower molar, I would then, after having opened up the pulp cavity, promote suppuration instead of trying to prevent or subdue it; for which I recommend hot fomentations--a cloth squeezed out of hot water, and held to the face opposite the tooth--or tincture of aconite to the gum; but that should be used with discrimination as to *when* to use it, or its application may be worse than useless. In the treatment of periostitis, it should be used only when suppuration is probable or inevitable. The primary stage of periostitis seems to me to require treatment of a very opposite kind.

To a tooth in this state, as also before suppuration has taken place, we should do no more at the first sitting than endeavor to relieve the pain. The proper cleaning of the canals should not be attempted for a day or two after, or for three or four days, if there is any swelling of the face and difficulty in opening the mouth freely, and the patient should be directed to pick the food out of the cavity in the tooth after each meal. I have never found much good from a leech to the gum at any stage of inflammation. Lancing, when it is necessary, I have found do more good and is more easily and quickly done. Apply chloroform to the gum (or the new anæsthetic, cocaine), and it can be lanced freely with but little pain to the patient.

Allow me to digress for a moment, and give very briefly my experience of cocaine. I used a 20 per cent. solution of the hydrochlyrate, and for lancing the gum or for extracting teeth I have found it no better than chloroform applied to the gum. The anæsthesia seems to be very superficial, but, nevertheless, decided. For excavating sensitive dentine, it is to be highly commended. For that purpose I have been using for some time the oxide of calcium, left in tooth till the following day, and have been much pleased with it; but here we have an agent superior, which permits the completion of the excavating at the first sitting.

The extraction of teeth, in order to fill the canals, and then replanting them, I cannot discuss. I never practiced it, and I never agreed with it in theory.

We will now consider the cleaning and preparation *for filling of the nerve canals*, which should be done as soon as the tooth is quite free from pain. The cavity of decay should be so shaped, that the canals be made easy of access. If the decay has not made it so, then it must be shaped with chisel, file, and burring engine, and it is often necessary to cut away a great part of the crown of the tooth.

In large posterio-approximal cavities of second bicuspids, and all the molars, the cavity should be opened up to nearly the middle of the crown, making it resemble a compound one. Most of this would be done with the chisel, and finished, shows the shape of cavity I am describing. With *small* posterio-approximal cavities, it is often better to open through with the file, putting it between the teeth and holding it while filing at an obtuse angle, that is, making something like a broad wedged-shaped opening between the teeth.

Sometimes, in first molars, and very often in bicuspids, we can get sufficiently good access to the cavity and canals by cutting away the posterio-buccal surface of the tooth, as is shown in this upper molar. But where we have to fill canals with the aid of the mouth mirror, it is usually better to cut both buccal and lingual sides. Having enlarged the cavity of decay, we then open up the pulp chamber with burring engine and excavator, and then enlarge the orifices of the canals, making a kind of trumpet-mouth shape to each of them. In molar teeth, with a buccal cavity, small, and near the gum, the only thing we can do is to make a crown cavity with the burring engine, as shown in this lower molar. With a drill such as I now exhibit, I make two, three, or four holes through the crown to the pulp chamber, and then open up with a burr head, usually a long burr drill. I consider a case like this a difficult one, and if the patient were over middle age I might hesitate to attempt the treatment of it, but in young teeth it can be successfully accomplished.

You have heard the saying "keep your powder dry." The equivalent of that to us should be "keep your instruments sharp and well tempered." I am certain there are cases of failure when there need not be, because that is not attended to. To clean the canals, I usually begin with bicarbonate of soda. I fill the cavity of decay with it, add one drop of water from a syringe, and with one of these canal needles, slightly roughened, work it up and down and against the sides of the canals, then syringe out with warm water. If the canals are very foul, I do that twice or more. I then put one crystal of permanganate of potassium in the cavity of decay, add one drop of water, and work it into the canals as I did the soda, then syringe out well. Care must be taken not to force any of it through the apical foramen, because if any go through it will give pain, lasting sometimes two or three hours. I then fill the canals with carbolic acid, forcing it well to the very ends in the manner which I will now describe. Saturate well with carbolic acid a small pellet of cotton wool, put it in the cavity of decay, pass a *smooth* needle by the side of the wool into the canal and work it up and down. The needle, if it is of a proper thickness, acts as a piston, and the liquid can be forced to the very ends of the canals. I usually begin with a thick needle and finish with one fine enough to force the liquid to the very end, and if a little go through the foramen so much the better. When applying it to the roots of front teeth where the crown has been lost by decay, or has been cut off preparatory to inserting artificial teeth, having no cavity to hold the cotton wool, I wrap a very slight shred of wool or floss silk round a slightly roughened needle and keep dipping it in the acid, and forcing it in the canal till it is filled. Having cleaned the canals, and applied carbolic acid, that is enough for the second visit. At the third visit, if the canals are clean and the tooth seems well, I use bicarbonate of soda and carbolic acid as before, and then fill the root and the tooth. Permanganate of potassium should not be used at the last visit, because it discolors the tooth, and if there is still any odor from the tooth, it should not be filled, but should be treated with soda and the permanganate as before, finally filling the canals with soda and closing up with wax or cotton wool, with instructions to the patient to remove the wool if the tooth should ache. My usual mode of testing the cleanliness of a canal is to pass one of these needles into it, then withdraw the needle and smell it. Whether the putrid matter enters the dentinal tubules to any great extent I do not know, but occasionally we find a tooth so permeated with this foul odor that it requires more time to purify it, and it is sometimes at a fourth or even a fifth visit that I fill such a tooth, and as long as there is the slightest trace of pus in the canals, or fistulous opening on the gum or gumboil, the treatment should be continued with soda and carbolic acid, using the latter very freely. Sometimes about one-third of a nerve remains alive at the end of a root, very tenacious of its vitality, and requiring patience to overcome it, but persistent acupuncture with carbolic acid will do it. I have not yet tried cocaine for this purpose, but would hope for good results from it.

We hear of some operators drilling through gum and bone and scraping off a pus forming sac from the end of a root, but such treatment seems to me a little too heroic and unnecessary. Excepting in aged teeth carbolic acid can usually be forced through the apical foramen, and I feel assured that when persistently applied, a very small quantity at a time of the liquid, full strength, is enough to destroy the sac or it brings it into a state of quiescence. When it is possible the foramen should be slightly enlarged where there is a persistent discharge of pus. The treatment to be quite successful should be continuous, and when we have a patient who does not come at the appointed times, or comes only when the tooth has begun to ache again, it is better to advise the extraction of the tooth. I have got myself into disgrace by attempting to treat such a tooth for such a patient.

I fill roots always with oxychloride of zinc and nothing else, and I do not intend to discuss the use of any other material for that purpose. Suffice it to say that some years ago I used cotton wool, gutta percha, gold, amalgam, etc., but I have been most successful with oxychloride of zinc. The best oxychloride I have found for the purpose is Guillois' cement, because it is slow setting. The method of filling is to mix it a very little thicker than cream, put it in the cavity of decay, introduce a thick needle into the canal, and pump the oxychloride as far as the thick needle will carry it, then use a thin needle to carry it to the end of the canal. It should be well and freely forced to the very end, and as soon as it *begins* to set it should be left alone till hard. Then scrape the oxychloride out of the cavity of the tooth, and fill with whatever material is most suitable for it. In filling roots where the whole of the crown is gone, I fill two-thirds with oxychloride and the remaining third with amalgam. In filling the canals of posterio-approximal cavities in molars, where it has to be done with the mouth-mirror, if more time is wanted in working the oxychloride, it can be gained by adding about 20 per cent. of water to the liquid, which makes it set slower.

It has been said that, in filling canals with oxychloride of zinc, we should be careful not to force it through the foramina, but there can be no fear of harm from that when we do not enlarge them. They are so small that very little can go through, even though there were a vacuum in the socket to receive it, which there is not. Besides, it is a liquid we are using and not a solid substance, which might be forced into the socket with pressure. Further, I maintain that a little forced through—if it be only a little—it will do no harm beyond giving slight pain for half an hour or an hour.

Dr. Marshall Webb has said (Notes on Operative Dentistry, 1883) we should enlarge the canal, when possible, up to the foramen, and then, before filling with oxychloride of zinc, close the foramen with gold foil. It seems to me that in doing so, we would run a risk of having the gold foil pressed either too far or not far enough, either of which two conditions would be dangerous to the success of the operation.

Dr. J. Foster Flagg says (Dental Pathology and Therapeutics, 1873) that if one-third of the root be left unfilled, sooner or later it will give trouble. I think that is true of some roots only. I would not like to leave an upper incisor root only two-thirds filled, but I have done so often with the buccal roots of upper molars, and I know that many of these teeth have been all right for several years.

I think it is not good practice to fill roots with cotton wool and creosote, or carbolic acid. I remember three cases of periostitis, quite lately, where I found, on removing the stopping, the canals filled with cotton, and smelling as strongly of creosote as if newly put in. Although the roots in these cases were apparently clean, trouble had been set up, from a few weeks to a few months, after filling.

I will give you here, in their order, cavities of decay in teeth, which I find easy or difficult of access to the canals beginning with the easiest:—

Upper front teeth—approximal and lingual cavities.

Upper and lower bicuspids and lower canines—approximal and crown cavities.
Upper front teeth—labial cavities.
Lower incisors—approximal cavities.
Upper molars—crown and antero-approximal cavities.
Lower molars— do.
Upper molars—posterio-approximal cavities.
Lower molars-- do.
Upper and lower molars--buccal and lingual cavities.

One word about temporary teeth. The difficulty in treating the roots of temporary teeth consists only in the age of the patient. The same teeth in the mouth of an adult could be easily treated. Appointments are also more likely to be neglected —very often after the first visit--when toothache has been relieved, we see no more of the patient till toothache has returned and the tooth probably in a much worse state than before. Thickening of the membrane and congestion at the root seems to come on sooner than with permanent teeth, probably because the foramina are often wide.

I only attempt to treat those in which the canals are easy of access. We can hardly do more for a patient so young, and usually restless, and little value is generally put on a tooth which is to fall out in a short time. When time for shedding arrives, absorption of the root does not take place as when in the normal state, and it is well to caution the patient's friends of that. If permanganate of potassium be used, it should be done with great care.

We cannot be successful in every case. I reckon my failures at about ten or fifteen per cent., and here is one that occurred to me three weeks ago. It is a lower bicuspid, with two distinct canals, and the lingual one, as will be seen, is filled to fully two-thirds of its length with secondary dentine, which was probably the cause of the failure.

Secondary dentine and pulp stones, especially the former, are sometimes a formidable barrier to the opening of a nerve canal. Excepting in straight roots, easily got at with a drill, when a canal is filled to nearly half of its length with secondary dentine, nothing can be done, and we have only to hope it will not cause any trouble.—*British Journal of Dental Science.*

